# Preparation of High-Energy Activated SiC Particles and Their Dispersion and Reaction Behavior in Hypoeutectic Gray Cast-Iron Melt

**DOI:** 10.3390/ma18235264

**Published:** 2025-11-21

**Authors:** Chunfeng Wang, Zhejun Li, Chuangang Huang, Runze Li, Qingyan Liang, Kebin Li, Jie Hu, Feng Jiang

**Affiliations:** 1 School of Intelligent Welding Technology, Guangxi Technological College of Machinery and Electricity, Nanning 530007, China; wangchunfeng@gxcme.edu.cn; 2 School of Mechanical and Automotive, Guangxi University of Science and Technology, Liuzhou 545616, China; 18161019311@163.com (Z.L.); 19507762040@163.com (R.L.); 15934894716@163.com (K.L.); 100001086@gxust.edu.cn (F.J.); 3 Guangxi Yuchai Machinery Co., Ltd., Yulin 537005, China; 19167041127@163.com

**Keywords:** hypoeutectic gray cast iron, high-energy activation, silicon carbide, degree of supercooling, latent heat of crystallization

## Abstract

This study addresses the issues of coarse primary austenite dendrites and uneven graphite distribution in hypoeutectic gray cast iron. High-energy mechanical activation technology was used to prepare high-energy activated SiC particles (EASiCp), and the regulatory mechanisms of trace additions (0–0.15 wt.%) on the solidification process and microstructure properties of hypoeutectic gray cast iron were systematically investigated. The results indicate that high-energy activation treatment reduced the average particle size of SiC particles from 26.53 μm to 9.51 μm and increased their specific surface area from 0.35 m^2^/g to 1.78 m^2^/g. X-ray diffraction (XRD) analysis revealed that the grain size was refined from 55.5 nm to 17.4 nm, with significant lattice distortion. The absorption rate of EASiCp in the melt stabilized between 68–72%, with particles predominantly dispersed within the grains (78.12%) and at grain boundaries (21.88%) in sizes ranging from 0.3 to 2 μm. The addition of EASiCp enhanced the solidification undercooling from 5.3 °C to 8.4 °C and reduced the latent heat of crystallization from 162.6 J/g to 99.96 J/g due to its endothermic reaction in the melt (SiC + Fe → FeSi + C) and heterogeneous nucleation effects. In terms of microstructure, the addition of 0.15 wt.% EASiCp increased the primary austenite dendrite content by 35.29%, reduced the secondary dendrite arm spacing by 57.98%, shortened the graphite length from 0.46 mm to 0.20 mm, and refined the eutectic colony size from over 500 μm to 180 μm. The final material achieved a tensile strength of 308 MPa, an improvement of 12.82% compared to the unadded group. Mechanistic analysis showed that EASiCp facilitated direct nucleation, reaction-induced “micro-area carbon enrichment,” and a synergistic effect in suppressing grain growth, thereby optimizing the solidification microstructure and enhancing performance. This study provides a new method for the efficient nucleation control of hypoeutectic gray cast iron.

## 1. Introduction

Gray cast iron holds an irreplaceable position in critical components such as automotive engine blocks and machine tool guideways due to its excellent casting properties, cost-effectiveness, and damping characteristics [1]. Hypoeutectic gray cast iron, a key subclass of gray cast iron, features a carbon content below the carbon equivalent corresponding to the eutectic point [2]. During solidification, primary austenite precipitates first, growing in dendritic morphology; as temperature decreases further, the remaining liquid undergoes eutectic transformation to form a eutectic structure consisting of graphite and austenite [3]. Following primary crystallization, austenite undergoes eutectoid transformation, yielding a matrix of pearlite or ferrite, within which lamellar graphite is distributed. While graphite imposes some fragmentation on the matrix, it endows the material with unique properties. Hypoeutectic gray cast iron inherits the advantages of gray cast iron, including excellent vibration damping, superior machinability, and low cost, while exhibiting higher strength than hypereutectic gray cast iron due to the presence of primary austenite dendrites, making it suitable for moderate load conditions [4]. Its performance is highly dependent on the morphology of primary austenite dendrites, graphite morphology, and eutectic cell structure formed during solidification. However, conventional hypoeutectic gray cast iron often suffers from coarse primary austenite dendrites and non-uniform distribution of graphite flakes during solidification, leading to insufficient material strength and significant fluctuations in mechanical properties [5,6,7].

Modifying the microstructure of hypoeutectic gray cast iron through carbon equivalent (CE) adjustment or inoculant addition (e.g., ferrosilicon, FeSi) represents an effective strategy, which fundamentally addresses issues such as undesirable graphite morphology, inhomogeneous matrix structure, and excessive harmful phases through systematic regulation of both macroscale compositional optimization and microscale nucleation intervention [8,9,10]. Adjusting CE—a critical parameter integrating the combined effects of carbon, silicon, and other elements on graphite formation in cast iron—alters the graphitization driving force of molten iron [11]. When CE approaches the eutectic composition, the undercooling required for graphite precipitation decreases, curbing the excessive growth of primary austenite dendrites and facilitating the formation of uniform graphite-austenite eutectic structures during the eutectic transformation [12]. This process concurrently suppresses cementite formation and prevents chill structures. Additionally, CE indirectly influences subsequent eutectoid transformations by modulating the synergistic effects of carbon and silicon to balance the pearlite-to-ferrite ratio in the matrix, thereby optimizing the trade-off between strength and toughness while mitigating structural defects caused by compositional segregation [13]. Inoculation, exemplified by FeSi addition, primarily acts through microscale nucleation control. FeSi introduces heterogeneous nucleation sites with high lattice matching to graphite, activating dormant nucleation substrates in the molten iron and significantly increasing graphite nucleation density [14]. This leads to refined graphite size and eutectic cell structure, avoiding coarse or abnormal graphite morphologies. However, the development of efficient and controllable external nucleation technologies to further optimize solidification processes remains a focal point in both academic research and industrial practice.

In recent years, silicon carbide (SiC) has been regarded as a potential inoculant for cast iron due to its unique physicochemical properties, such as a high melting point and low lattice mismatch with graphite lattice. Experimental evidence indicates that SiC undergoes decomposition in molten iron (SiC → Si + C), which not only replenishes carbon sources but also promotes heterogeneous nucleation through the surfaces of undissolved particles [15,16]. Krzysztof Janerka et al. [17] found that in eutectic ductile cast iron systems, increasing SiC addition decreases the liquidus temperature while maintaining a stable solidus temperature, narrowing the crystallization temperature range and thereby improving the quantity and quality of graphite precipitation. Additionally, the graphite nodules exhibit an “irregular morphology with protruding lamellae” and increased internal microcracks after SiC addition. B. Domeij et al. [18] further demonstrated that SiC promotes early uniform dendrite growth by providing heterogeneous nucleation sites, reducing the undercooling required for austenite nucleation. However, existing studies still exhibit multiple gaps that hinder the efficient regulation of hypoeutectic gray cast iron. First, for the ultrafine and highly active SiC particles (SiCp) subjected to high-energy activation treatment, their dispersion behavior, absorption rate, and particle-size-dependent dissolution kinetics in the hypoeutectic gray cast iron melt remain unclear. Second, the quantitative influence laws of such activated particles on the thermodynamic and kinetic parameters of the solidification process—such as undercooling and crystallization latent heat—as well as the structure–property correlations governing the resultant microstructural refinement, have not yet been systematically elucidated. Finally, the microscopic mechanism by which high-energy activated SiC particles (EASiCp) regulate the primary austenite dendrites and eutectic clusters through the synergistic effects of “direct nucleation” and “reaction-induced carbon-rich zone formation” remains to be deeply clarified. In particular, the quantitative correlation model between this microstructural evolution and the resulting mechanical properties (e.g., tensile strength and hardness) has not yet been established, which hinders the transformation of this technology from a laboratory phenomenon into industrial application.

To address the aforementioned research gaps, this study employs high-energy mechanical activation technology to prepare SiCp, and systematically investigates their role in the solidification process of hypoeutectic gray cast iron through a combination of experimental research and theoretical analysis. The focus of this work includes: (1) Accurate characterization of the dispersion behavior and reaction mechanisms of EASiCp within the molten alloy; (2) Quantitative analysis of their influence on key solidification kinetic parameters, such as undercooling and latent heat of crystallization; (3) In-depth elucidation of the microscopic mechanism by which EASiCp achieve simultaneous refinement of primary austenite dendrites, graphite, and eutectic clusters through a “nucleation promotion–growth inhibition” synergistic mechanism. Ultimately, this study aims to establish a comprehensive quantitative correlation model linking process parameters → solidification kinetics → microstructure → tensile strength, thereby providing both the theoretical foundation and technical support for the development of high-efficiency and controllable nucleation technologies for high-performance cast iron.

## 2. Experimental Materials and Methods

### 2.1. Selection of Raw Materials

The EASiCp used in the experiment was primarily composed of α-SiC, sourced from China National Pharmaceutical Group Chemical Reagents Co., Ltd. (Shanghai, China). Its key parameters are shown in Table 1, with these data compiled from the manufacturer’s provided datasheet and the GB/T 2480-2008 standard [19].

The hypoeutectic gray cast iron matrix was melted using 50 wt.% scrap steel, 35 wt.% re-melted iron, and 15 wt.% pig iron, with a target carbon equivalent (CE = C% + Si%/3) + (S + P)%/2) of 3.8–4.0 wt.%. The basic chemical composition is provided in Table 2. In the experiment, a SiCa-Ba inoculant at 0.4 wt.% (relative to the melt mass) was placed at the bottom of the crucible for inoculation treatment.

### 2.2. Preparation of Experimental Samples

#### 2.2.1. Preparation of EASiCP

EASiCp was prepared using an HVM-1 self-vortex, medium-free high-energy milling machine (AGOII type planetary ball mill; Institute of Mechanochemistry of Russia, Novosibirsk, Russia). The equipment specifications are as follows: rotor diameter of 525 ± 5 mm, centrifugal acceleration of 100× *g*, generating a multi-stage vortex flow field with an average flow velocity of 12.5 m/s and an impact force up to 100 g. The treatment duration was 30 min, enabling the micronization and nanocrystallization of the α-SiC raw material. For the determination of EASiCp absorption rate, a specially prepared batch containing 0.1 wt.% nickel (Ni) as a tracer element was used, while all other experiments employed Ni-free EASiCp.

#### 2.2.2. Preparation of Hypoeutectic Gray Cast Iron Samples with EASiCP

The hypoeutectic gray cast iron was melted in a 500 kg capacity coreless medium-frequency induction furnace (Furnace #1). When the melt temperature reached 1450–1470 °C, 100 kg of molten iron was poured into a 150 kg ladle that had 0.4 wt.% SiCa-Ba inoculant pre-loaded. The melt was then poured at a temperature of 1410–1430 °C into φ30 mm × 200 mm test rods, which served as the control group without EASiCp addition.

For the preparation of EASiCp-modified test rods, the additions were 0.05, 0.10, and 0.15 wt.%. EASiCp was tightly wrapped in aluminum foil at a rate of 10–15 g per packet. One hundred kilograms of molten iron were transferred to a 150 kg coreless medium-frequency induction furnace (Furnace #2). When approximately 10 kg of molten iron had been poured, the pre-set amount of EASiCp was added, and the remaining molten iron was then poured in. Using the electromagnetic stirring function of the medium-frequency furnace (in vortex mode, with a heating rate of 100 °C/min), after about 30 s, when the iron temperature reached 1450–1470 °C, the entire melt was poured out and cast using the same inoculation and pouring process as the reference sample. The chemical composition of the iron melt before pouring for each sample was analyzed using a direct-reading spectrometer. Any lost elements were supplemented to ensure composition consistency, with the final measured composition shown in Table 3.

### 2.3. Testing and Characterization

#### 2.3.1. Particle Size and Crystal Structure Analysis

Particle Size Distribution: The particle size distribution was measured using a Malvern Mastersizer 2000 laser particle size analyzer from Malvern Instruments Ltd., Malvern, Worcestershire, UK. The measurement range was 0.02–2000 μm, with a scanning frequency of 1000 times per second. Each sample was tested in triplicate, and the average value was taken. Subsequently, based on the particle size distribution data, the specific surface area of the particles was calculated using the instrument’s built-in software v5.12C (based on a spherical model).

Crystalline Structure: The crystalline structure was analyzed using a Panalytical Empyrean X-ray diffractometer (XRD) from Panalytical B.V., Almelo, Netherlands. The instrument used a Cu target with a working voltage of 40 kV and a current of 40 mA. The scanning range was 20–100°, with a step size of 0.02°. The average grain size was calculated using the Scherrer formula [20]:

(1) $$D=\frac{K\lambda }{\beta \cos\theta }$$

In the formula, *K* represents the Scherrer constant, which is taken as 0.89 here, and *λ* represents the X-ray wavelength, which is taken as 0.154 nm here; where *β* refers to the full width at half maximum and *θ* refers to the diffraction angle. To ensure the statistical representativeness of the results, this study selected the three main diffraction peaks—(111), (220), and (311)—and calculated their values, taking the average of the results.

#### 2.3.2. Microstructure Observation

Scanning Electron Microscope (SEM) and Energy Dispersive Spectrometer (EDS): The macro-distribution of EASiCp was observed using a Zeiss SUPRA 55 field-emission SEM from Carl Zeiss AG, Oberkochen, Germany. The acceleration voltage was set between 0.02–30 kV, and secondary electron imaging (SEI) mode was used to locate the EASiCp distribution. EDS was employed for point scanning (quantitative composition analysis), line scanning (interface element variations), and surface scanning (element spatial distribution). A statistical verification was carried out for ≥300 suspected EASiCp particles, excluding interference from impurity phases such as MnS or FeSi/Fe_3_C.

Optical Microscope (OM): Microstructure observations were conducted using a Zeiss optical microscope from Carl Zeiss AG, Oberkochen, Germany. For matrix structure observation, the samples were etched using 4% nitric acid alcohol. To observe primary austenite dendrites, the samples were heated in the furnace to 860 °C, held for 30 min, then furnace-cooled at a rate of 40 °C/h to 600 °C, followed by air cooling to room temperature and polishing.

#### 2.3.3. Solidification Kinetics Analysis

Differential Scanning Calorimetry (DSC) Analysis: The solidification temperature curve and crystallization latent heat were measured using a Netzsch STA449 simultaneous thermal analyzer from Netzsch GmbH, Selb, Bavaria, Germany.

Test Conditions: The temperature range was 0–1350 °C, with a heating and cooling rate of 10 K/min. The sample mass was approximately 25 ± 0.5 mg. An Al_2_O_3_ crucible was used under a high-purity argon atmosphere (flow rate 50 mL/min).

The instrument was calibrated using high-purity indium (In) as the standard sample. The degree of undercooling (Δ*T*) was calculated according to the formula:

(2) $$\Delta T=T_{ic}-T_{p}$$

where *T_ic_* is the extrapolated crystallization onset temperature and *T_p_* is the peak temperature. Each group of samples was tested three times, and baseline correction of the DSC curves was performed using the tangent method.

#### 2.3.4. Mechanical Properties Testing

The mechanical properties were evaluated through room-temperature tensile tests using a CMT5205 microcomputer-controlled electronic universal testing machine from Shenzhen SANS Materials Testing Co., Ltd. (also known as Shenzhen SANS Metrology Technology Co., Ltd.; CMT), Shenzhen, Guangdong Province, China. The specimens were prepared in accordance with the GB/T 228.1–2010 standard [21], with a diameter of 20 mm, a gauge length of 100 mm, and a tensile rate of 6 mm/min. Hardness testing was conducted using an HB-3000 Brinell hardness tester from Dongguan Huayin Test Instrument Co., Ltd., Dongguan, Guangdong Province, China, featuring a measurement range of 8–450 HBS, a maximum allowable specimen height of 230 mm, a distance of 120 mm between the indenter center and the machine wall, and a power supply of AC 380 V. For each testing group, three specimens were measured, and the average value was reported.

## 3. Results and Discussion

### 3.1. Characteristics of EASiCp

#### 3.1.1. Particle Size Distribution Analysis

**Figure 1:**
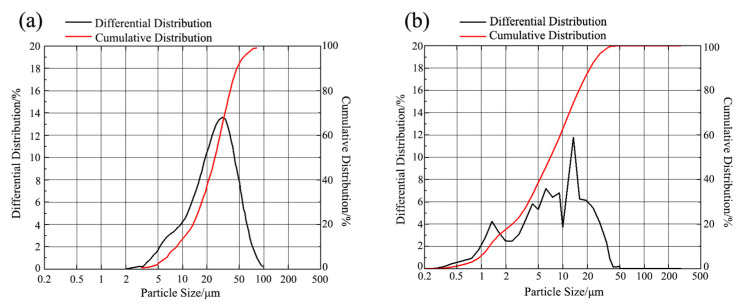
Particle Size Distribution Diagram of EASiCp ((**a**) Before Treatment; (**b**) After Treatment).

The particle size distribution of reinforcement particles significantly affects their behavior in the melt and is crucial for analyzing the strengthening mechanism. To investigate the effect of high-energy activation treatment on the size of SiCp, both pre- and post-treatment SiCp were tested and analyzed. Based on the laser particle size distribution data and using the spherical particle model, the specific surface area was calculated (Note: This calculated value is primarily used to characterize the relative change in the specific surface area of the particles before and after high-energy activation treatment, serving as an effective means to evaluate the mechanical activation effect; the measurement repeatability error is less than ±2%), and the results are shown in Table 4 and Figure 1. Furthermore, Table 5 presents the specific data on the particle size distribution of EASiCp after high-energy activation treatment.

As shown in Table 4, the high-energy activation treatment significantly refined the SiCp: the average particle size decreased from 26.53 μm to 9.51 μm, and the specific surface area increased from 0.35 m^2^/g to 1.78 m^2^/g. This indicates a significant increase in the atomic activity on the particle surface, which is crucial for analyzing the behavior of EASiCp in the gray cast iron melt. Figure 1 shows that after high-energy activation treatment, 38.54% of the SiCp were below 5 μm in size. Among them, 4.68% were smaller than 1 μm, 12.8% were between 1 μm and 2 μm, and 21.06% were between 2 μm and 5 μm. This particle size range is favorable for heterogeneous nucleation [22].

Notably, as shown in Figure 1b, after high-energy activation treatment, the particle size distribution curve of SiC particles exhibits a distinct non-monotonic “sawtooth” pattern, which significantly differs from a typical unimodal distribution (such as a log-normal distribution). This phenomenon indicates that the activated particle system presents a multimodal distribution, a result of the dynamic competition between “fracture” and “agglomeration/aggregation” during the high-energy mechanical activation process. On one hand, the intense mechanical impacts and shear forces are sufficient to effectively break down the original coarse particles, generating a large number of submicron-sized primary fine particles. On the other hand, these newly generated fine particles have extremely high surface energy and may undergo brief, weak agglomeration or aggregation during high-speed collisions, forming “secondary particles” of intermediate sizes. As a result, the final particle size distribution is a composite representation of various populations, including unbroken residual particles, secondary agglomerated particles, and primary fine particles. This complex, non-equilibrium state precisely demonstrates that the high-energy activation treatment successfully drives the particle system into a highly active state. From an application perspective, this wide particle size distribution, which includes both micron and submicron scales, may be more beneficial for forming functional complementarity within the cast iron melt, providing a richer size gradient and active sites for subsequent heterogeneous nucleation and reaction behavior within the melt.

#### 3.1.2. Crystal Structure

**Figure 2:**
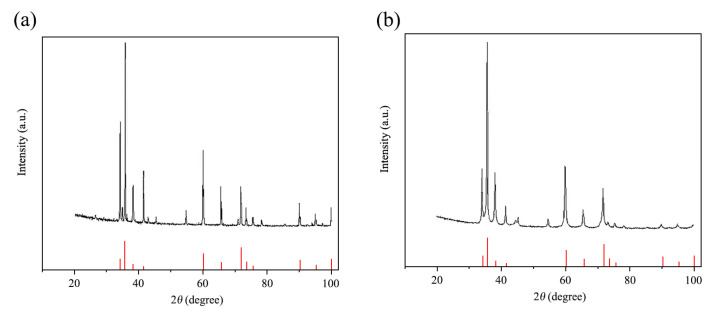
Comparison of XRD Patterns of SiCp Before and After High-Energy Activation Treatment ((**a**) Before Treatment; (**b**) After Treatment). Note: Red vertical lines: Standard peak positions of 6H-SiC (PDF #29-1131).

Figure 2 shows the XRD patterns of SiCp before and after high-energy activation treatment. The diffraction peaks of both samples match well with those of hexagonal α-SiC (International Centre for Diffraction Data (ICDD)—Powder Diffraction File (PDF) #29-1131), indicating that the high-energy activation did not change the crystal structure of SiC. However, the diffraction peaks of EASiCp show decreased intensity and peak broadening, suggesting grain refinement and lattice distortion of the particles.

To accurately quantify the degree of grain refinement and enhance the statistical reliability of the results, the Scherrer equation was applied to calculate the grain size from the three strongest and well-resolved diffraction peaks—(111), (220), and (311)—with their average value taken as the final result. The results indicate that high-energy activation treatment reduced the average grain size of SiC from 46.9 nm (before treatment) to 15.8 nm (after treatment), representing a decrease of 66.3%. This significant refinement, confirmed at the nanoscale, aligns with the macroscopic refinement revealed by laser granulometry (where the average particle size decreased from 26.53 μm to 9.51 μm), collectively demonstrating that the high-energy activation technique achieved a synergistic refinement effect, extending from the microscopic grain level to the macroscopic particle scale.

### 3.2. Behavior of EASiCp in Hypoeutectic Gray Iron Melt

#### 3.2.1. Retention Rate of EASiCp

Ni was used as a tracer element to calculate the absorption rate of EASiCp, with the results shown in Table 6. When the EASiCp content ranged from 0.2–5 wt.%, the absorption rate remained stable at 68–72%, indicating that EASiCp has good compatibility with the hypoeutectic gray cast iron melt [16].

#### 3.2.2. Microscopic Dispersion State of EASiCp

**Figure 3:**
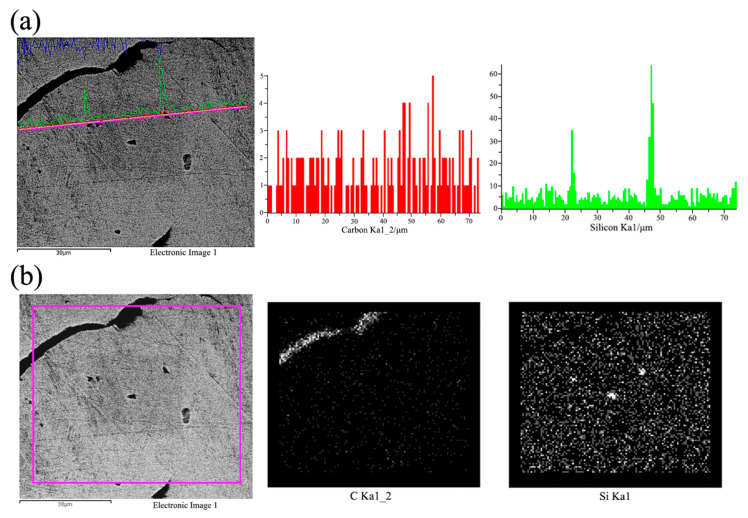
EDS Analysis of SiCp in the Sample ((**a**) Line Scan Analysis of the Interface Between SiCp and Iron Matrix; (**b**) Elemental Mapping of the Region Corresponding to (**a**)).

Figure 3 presents the SEM-SEI images, EDS line scan, and area scan results of EASiCp in hypoeutectic gray cast iron. In Figure 3a, it can be observed that the signals for Si and C are strongest within the particles, with a sharp decline at the interface, which corresponds perfectly with a sharp increase in the Fe signal. The steep change in element distribution at the interface suggests that no stable FeSi or Fe_3_C reaction layer forms between the SiCp and the iron matrix. This implies that the reaction (SiC + Fe → FeSi + C) is likely a non-equilibrium process, and the generated C rapidly diffuses into the surrounding melt, preventing the reaction products from stably existing at the interface. Figure 3b shows the elemental area scan distribution for the corresponding region. The results clearly indicate that the observed particles are regions of co-enrichment of Si and C, with the distributions of both elements highly overlapping, while the Fe signal is significantly reduced in this region. This confirms, in two-dimensional space, that these particles are primarily SiC.

To further confirm the particle composition, EDS point analyses were conducted on multiple particles similar to those shown in Figure 3a. The compositional results for one representative analysis point (Spectrum 44) are listed in Table 7. The results show that the combined atomic percentage of Si and C exceeds 70%, with an atomic ratio close to 1:2.4. Considering that EDS has certain quantitative errors in detecting ultra-light elements like C, this result is consistent with the stoichiometric characteristics of SiC. The detected Fe signal primarily arises from the matrix fluorescence effect, which is inevitable when the electron beam interacts with submicron-sized SiCp. Statistical analyses of more than 50 particles revealed similar characteristics, with no Fe-dominated phases detected. This rules out the possibility that these particles are large amounts of FeSi or Fe_3_C reaction products.

**Figure 4:**
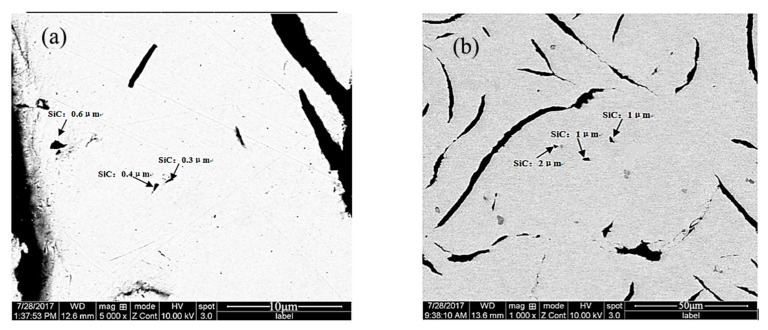
Distribution of SiCp in the sample ((**a**) 10,000×; (**b**) 2000×) (BSE).

**Figure 5:**
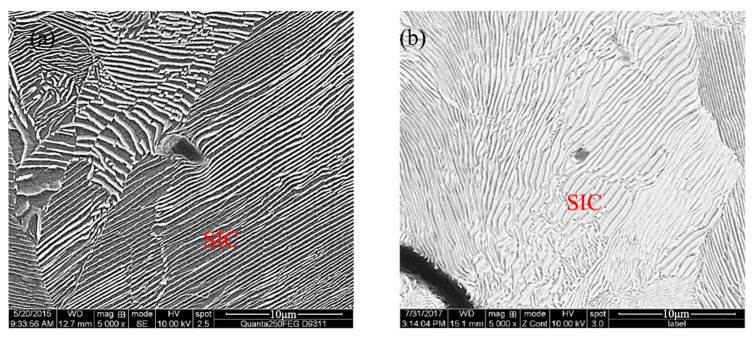
Distribution of SiCp in the sample ((**a**) locate at grain boundaries (SE); (**b**) dispersed within grains (BSE)).

Figure 4 shows the particle size distribution of SiC in the sample, and Figure 5 displays the morphology and distribution of SiC in the sample. It can be observed that the SiCp in the sample range in size from approximately 0.3–2 μm, and are dispersed within the pearlite grains or at the grain boundaries without any aggregation. This dispersion is primarily attributed to the vortex stirring of the molten iron during sample preparation, which facilitates the dispersion of SiCp.

**Figure 6:**
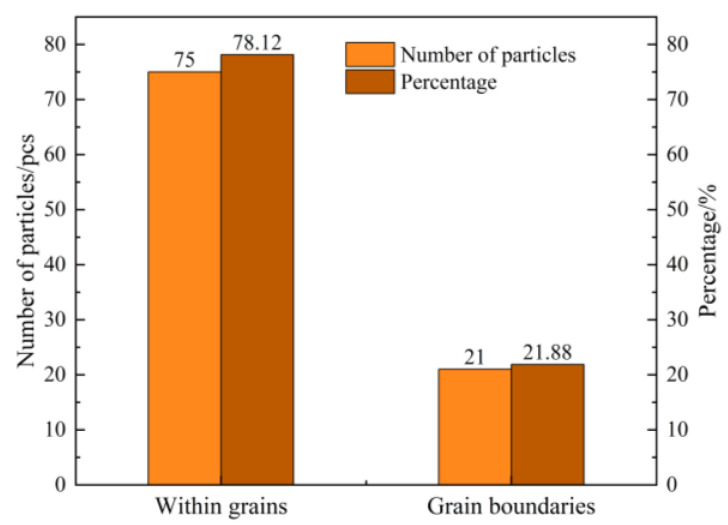
Distribution of 96 SiCp Particles.

**Figure 7:**
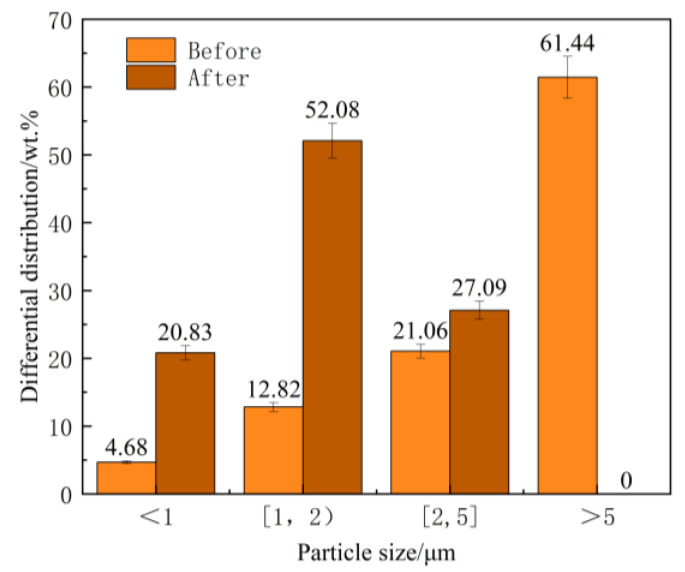
Particle size distribution of SiC powder before and after adding molten iron (An error of 5%).

A total of 96 SiCp from 62 SEM fields of view were statistically analyzed to determine their spatial distribution and size characteristics. The measured particle size data were compared with the differential and cumulative particle size distributions of the SiCp prior to their addition to the cast iron melt, as presented in Table 4. The comparison results are illustrated in Figure 6 and Figure 7, respectively. As shown in Figure 6, approximately 78.12% of the SiCp are distributed within pearlite grains, while 21.88% are located along the pearlite grain boundaries. The particles residing inside the grains primarily contribute to Orowan strengthening and dislocation strengthening mechanisms. In contrast, those situated at the grain boundaries impede heat transfer during solidification and the diffusion of solutes ahead of the dendritic front, thereby restricting grain growth and promoting grain refinement strengthening.

As shown in Figure 7, there is a significant change in the particle size distribution of SiC before and after being added to the cast iron melt. Specifically, the proportion of particles larger than 5 μm decreases from 61.44 wt.% to 0.00 wt.%, while the proportion of particles smaller than 1 μm increases from 4.68 wt.% to 20.83 wt.%, representing a 345.09% increase. The proportion of particles in the 1–2 μm range increases from 12.82 wt.% to 52.08 wt.%, showing a 306.88% increase. Additionally, the proportion of particles in the 2–5 μm range rises from 21.06 wt.% to 27.09 wt.%, corresponding to a 28.63% increase.

From the above results, it can be seen that after the addition of EASiCp to the hypoeutectic gray cast iron melt, the proportion of smaller particles (below 5 μm) significantly increased. Moreover, the smaller the particle size, the greater the increase in proportion. This indicates that the SiCp size decreases after being added to the gray cast iron melt, which is related to its reactive behavior in the melt.

#### 3.2.3. Dissolution Kinetics of EASiCp

The dissolution rate of EASiCp is defined as:

(3) $$v=\frac{d_{0}-d_{t}}{t}$$

where *d*_0_ is the initial particle size, *d_t_* is the particle size after *t* minutes, and *t* is the reaction time, approximately 8 min, which is the time from the addition of EASiCp to the end of solidification. Table 8 shows the dissolution rates of EASiCp with different initial particle sizes. Larger particles (28–50 μm) have a higher dissolution rate (3.3–5.6 μm/min) compared to smaller particles (<5 μm, <0.5 μm/min), reflecting a size-dependent dissolution characteristic.

To clarify the dissolution control mechanism, the Schmidt number (*S*_c_) and Sherwood number (*S*_h_) were calculated as follows:

Schmidt number:

(4) $$S_{c}=\frac{\mu }{\rho D_{c}}$$

where *μ* = 5 × 10^−3^ Pa·s is the viscosity of the molten iron, *ρ* = 7.0 × 10^3^ kg/m^3^ is the density of the molten iron, and *D*_c_ = 1 × 10^−8^ m^2^/s is the diffusion coefficient of carbon. The calculated value is *S*_c_ ≈ 70.

The Sc value indicates that mass transfer is dominated by diffusion, while the *S*_h_ value (which is higher than the static condition *S*_h_ = 2) confirms that the eddy current stirring enhances convective mass transfer. This mechanism is consistent with the experimental results (larger particles having higher Reynolds numbers and dissolving faster). It should be noted that the size-dependent dissolution of EASiCp differs from the classic Lifshitz-Slyozov-Wagner (LSW) theory (which predicts uniform dissolution), due to the following reasons: (1) The lattice distortion induced by high-energy activation increases the surface reactivity of small particles. (2) The convective mass transfer enhanced by the eddy current stirring accelerates the dissolution of larger particles.

### 3.3. Regulation of EASiCp on the Solidification Kinetics of Hypoeutectic Gray Cast Iron

#### 3.3.1. Degree of Supercooling

**Figure 8:**
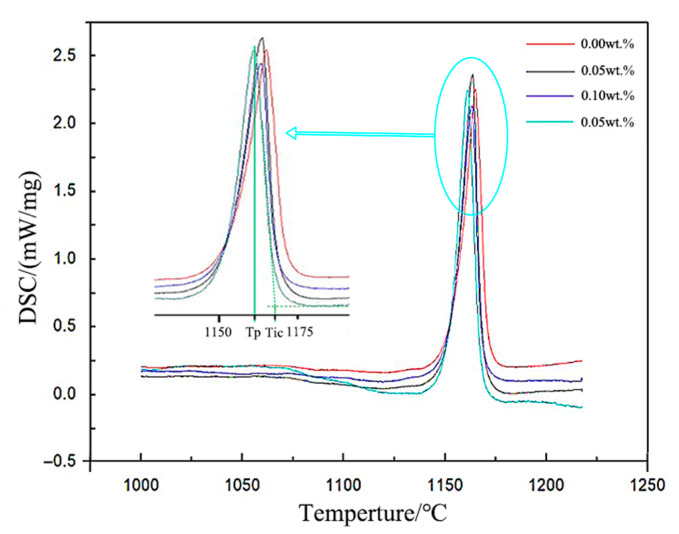
DSC Curves of Samples with Different EASiCp Addition Amounts.

**Figure 9:**
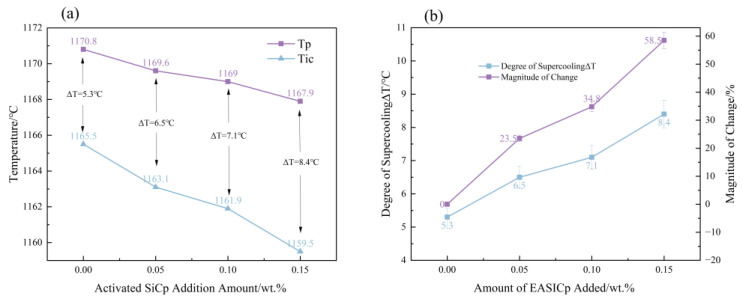
Effect of SiCp Addition Amount on Temperature and Degree of Supercooling During Solidification ((**a**) DSC Curves of Samples with Different EASiCp Addition Amounts, the temperature error is 5%; (**b**) Effect of EASiCp Addition Amount on Degree of Supercooling, An error of 5%).

Figure 8 and Figure 9 show the DSC (Differential Scanning Calorimetry) curves and undercooling trends of hypoeutectic gray cast iron with different additions of EASiCp. It can be observed that, with increasing EASiCp content: The extrapolated crystallization onset temperature (*T_ic_*) decreases from 1170.8 °C to 1167.9 °C; The peak temperature (*T_p_*) decreases from 1165.5 °C to 1159.5 °C; The undercooling (Δ*T*) increases from 5.3 °C to 8.4 °C, an increase of 58.5%. These results are consistent with the conclusions reported by A. I. Karlina, A. D. et al. [23]. The observed trend originates from the synergistic action of three factors:

(1) Thermodynamic effect: The dissolution of EASiCp increases the contents of C and Si, raising the carbon equivalent (CE) to 3.926. According to the liquidus line equation of hypoeutectic gray cast iron, an increase in CE lowers the theoretical crystallization temperature, resulting in a decrease in *T_ic_*.
(2) Kinetic effect: EASiCp provides more nucleation sites. The increase in active nucleation sites promotes simultaneous graphite nucleation at multiple locations, intensifying competition for carbon sources. Consequently, the decrease in *T_p_* is more pronounced than that in *T_ic_*.
(3) Endothermic effect: The reaction of SiC in the cast iron melt (reaction SiC + Fe → FeSi + C) is endothermic [24], which reduces the melt temperature and enhances the dispersion of crystallization.

According to classical nucleation theory, the nucleation rate is exponentially related to the square of the undercooling (*N*∝Δ*T*^2^ exp(−ΔG*/*kT*)). When the undercooling increases from 5.3 °C to 8.4 °C, the critical nucleation free energy (ΔG*) decreases by approximately 62%, significantly enhancing the thermodynamic driving force for heterogeneous nucleation. This change directly improves the nucleation efficiency of EASiCp, laying a crucial foundation for the subsequent refinement of the microstructure [25,26].

#### 3.3.2. Latent Heat of Crystallization

**Figure 10:**
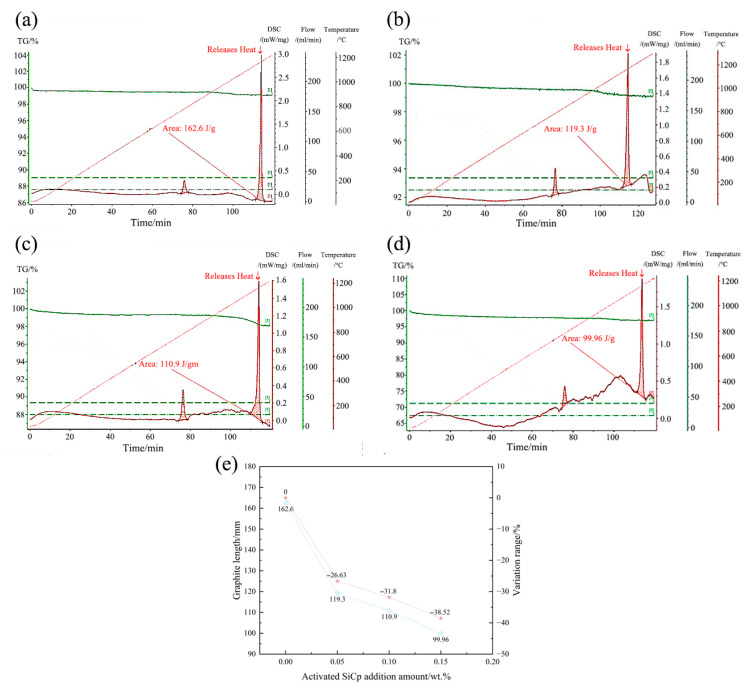
Effect of EASiCp Addition Amount on Latent Heat of Crystallization of Hypoeutectic Gray Cast Iron ((**a**) 0.00 wt.%; (**b**) 0.05 wt.%; (**c**) 0.10 wt.%; (**d**) 0.15 wt.%; (**e**) Variation Trend).

Figure 10 shows the latent heat of crystallization of hypoeutectic gray cast iron with different EASiCp additions. As the amount of EASiCp increases, the latent heat of crystallization decreases significantly: From 162.6 J/g at 0 wt.% to 119.3 J/g at 0.05 wt.% (a reduction of 26.63%); To 110.9 J/g at 0.10 wt.% (a reduction of 31.80%); To 99.96 J/g at 0.15 wt.% (a reduction of 38.52%).

The reduction in latent heat is primarily due to the sustained endothermic effect: the more EASiCp is added, the more heat is consumed by reactions during the solidification process, which macroscopically manifests as a decrease in the net latent heat released during the transition from liquid to solid. Control experiments confirm there are no other interfering factors: (1) The carbon equivalent is strictly controlled within the range of 3.876–3.926; (2) All groups use 0.4 wt.% SiCa-Ba inoculant; (3) Optical microscope observations show no free cementite, and the graphite is uniformly Type A.

The reduction in latent heat decreases the net heat supply during the dendritic growth stage, slowing the melt’s cooling rate and directly inhibiting the coarsening of primary austenite secondary dendrite arms (the distance between secondary dendrite arms is positively correlated with growth time and negatively correlated with cooling rate) [27]. Additionally, the dispersed exothermic heat makes the diffusion of carbon atoms during graphite growth more uniform, preventing local carbon enrichment that could lead to abnormal graphite growth, thus ensuring the shortening of graphite length.

### 3.4. Regulation of EASiCp on the Microstructure

The significant increase in undercooling (from 5.3 °C to 8.4 °C, a 58.5% increase) results in a dramatic enhancement of the nucleation driving force, which, in conjunction with the reduction in latent heat of crystallization (a 38.52% decrease), leads to a suppression of the growth rate. This creates a synergistic effect, breaking the original nucleation-growth equilibrium. The specific manifestation of this effect is the cooperative optimization of the primary austenite dendrites, graphite morphology, and eutectic structure [18,28].

Although directly observing the nucleation interface between SiC particles and primary austenite or graphite presents technical challenges—mainly due to phase transformations during the cooling process and the randomness of three-dimensional nucleation sites on two-dimensional sections—multiple indirect pieces of evidence strongly support the hypothesis that EASiCp acts as an effective nucleation core. First, the size distribution of EASiCp (primarily ranging from 0.3 to 2 μm) falls within the effective range for heterogeneous nucleation (typically 0.5 to 5 μm). Second, its spatial distribution statistics reveal that 78.12% of the particles are located within the grain, which strongly supports the mechanism of particles being captured as nucleation sites by growing dendrites. Furthermore, based on Bramfitt’s theory of planar misfit, preliminary calculations suggest a low lattice misfit (approximately 20%) between SiC and graphite, which provides theoretical feasibility for SiC to serve as a substrate for austenite nucleation. Similarly, a crystallographic matching relationship exists between SiC and graphite. Finally, as shown in Section 3.3, the introduction of EASiCp significantly increased the solidification undercooling, a typical characteristic of an increase in nucleation sites. These pieces of evidence collectively provide strong support for the role of EASiCp in promoting nucleation, offering a theoretical basis for the subsequent analysis of the evolution of the microstructure.

#### 3.4.1. Primary Austenite Dendrites

**Figure 11:**
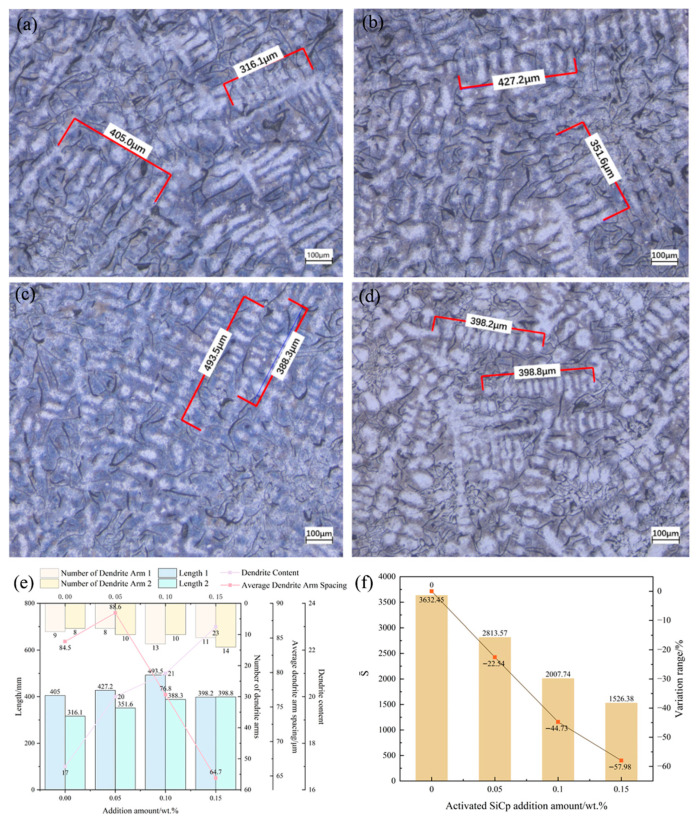
Effect of EASiCp Addition Amount on Primary Austenite Dendrites of Hypoeutectic Gray Cast Iron ((**a**) 0.00 wt.%; (**b**) 0.05 wt.%; (**c**) 0.10 wt.%; (**d**) 0.15 wt.%; (**e**) Variation of Average Primary Dendrite Arm Spacing; (**f**) Variation of “$\bar{S}$” Value).

Figure 11 shows the changes in primary austenite dendrites of hypoeutectic gray cast iron with different amounts of EASiCp added. It can be seen that all dendrites are equi-axed crystals, and as the EASiCp content increases: (1) The dendrite content increases from 17% to 23% (a 35.29% increase at 0.15 wt.%); (2) The secondary dendrite arm spacing (SDAS) decreases from 493.54 μm to 316.1 μm (a 57.98% decrease); (3) The dendrite morphology parameter $\bar{S}$ [29]:

(5) $$\bar{S}=\frac{A_{t}}{n_{t}}$$

where *A_t_* as the total dendrite area and *n_t_* as the number of dendrite cross-sections. This decreases from 3632.45 mm^2^ to 1526.38 mm^2^ (a 57.98% decrease), indicating a more developed dendritic branching.

The dendrite refinement is attributed to the promotion of heterogeneous nucleation by EASiCp and the increase in undercooling: (1) EASiCp particles smaller than 5 μm directly serve as nucleation cores (with a mismatch of approximately 20% with the austenite lattice); (2) The carbon-rich regions formed by the reaction of SiC in the cast iron melt (with a mismatch of about 4.9% with the austenite lattice) further increase the number of nucleation sites.

#### 3.4.2. Graphite Morphology

**Figure 12:**
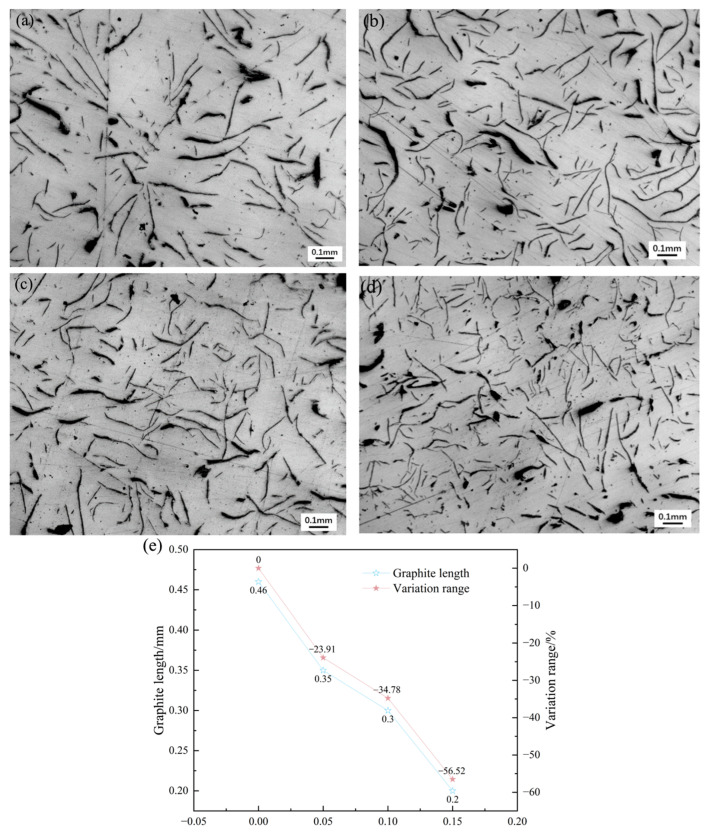
Effect of EASiCp Addition Amount on Graphite Morphology of Hypoeutectic Gray Cast Iron at Room Temperature ((**a**) 0.00 wt.%; (**b**) 0.05 wt.%; (**c**) 0.10 wt.%; (**d**) 0.15 wt.%; (**e**) Variation Trend).

Figure 12 shows the graphite morphology of hypoeutectic gray cast iron with different amounts of EASiCp added. It can be observed that all the graphite is of Type A, and as the EASiCp content increases: (1) The graphite length decreases from 0.46 mm to 0.20 mm (a 56.52% decrease); (2) The curvature of the graphite increases; (3) The graphite distribution becomes more uniform. A. I. Karlina et al. [23] also observed significant improvements in graphite morphology by adding a new type of modifier to gray cast iron.

The improvement in graphite morphology is attributed to: (1) The refinement of primary austenite dendrites, which limits the space available for graphite growth, causing the graphite to bend and branch upon contact with the dendrites; (2) The <5 μm EASiCp particles acting as nucleation cores for graphite (with a mismatch of approximately 12% with the graphite lattice), which increases the number of graphite particles and reduces their length.

#### 3.4.3. Eutectic Cells

**Figure 13:**
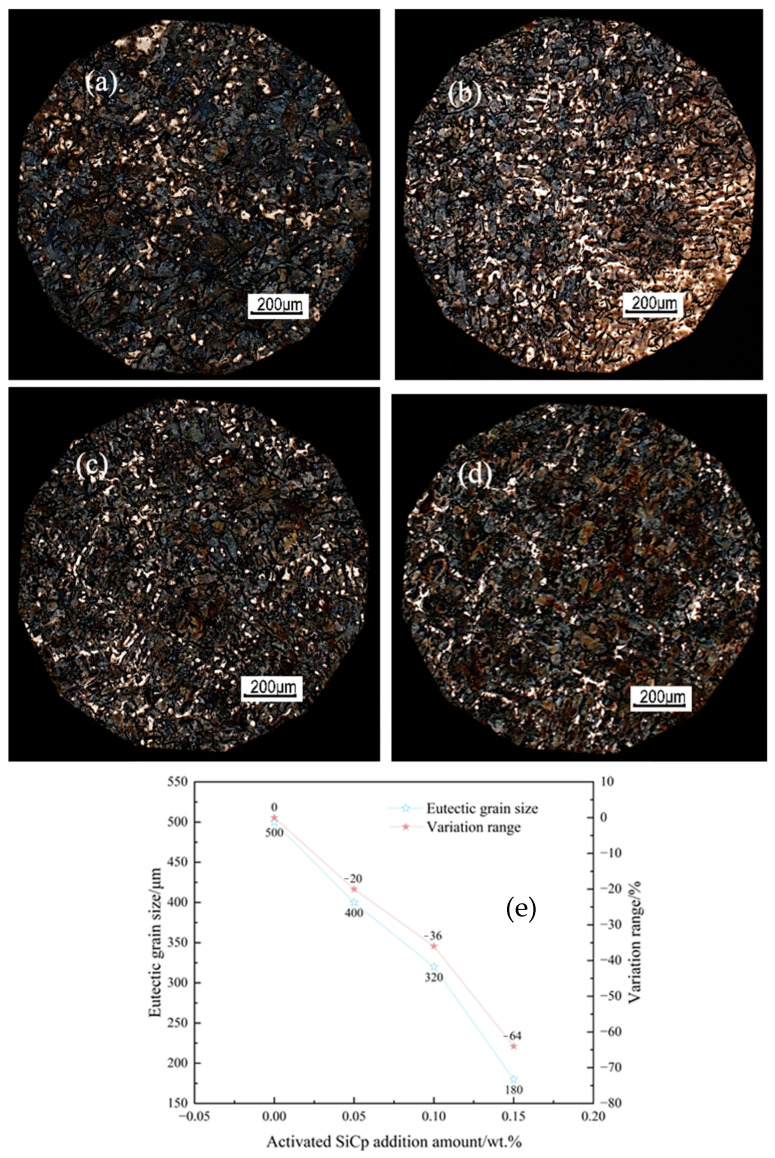
Effect of EASiCp Addition on Eutectic Cell Morphology of Hypoeutectic Gray Cast Iron ((**a**) 0.00 wt.%; (**b**) 0.05 wt.%; (**c**) 0.10 wt.%; (**d**) 0.15 wt.%; (**e**) Trend).

Figure 13 shows the eutectic structure morphology of hypoeutectic gray cast iron with different amounts of EASiCp added. As the EASiCp content increases: (1) The eutectic particle size decreases from above 500 μm to around 180 μm (a 64% decrease); (2) The number of eutectic particles increases significantly, and their size distribution becomes more uniform. Wang et al. [30] also achieved significant grain refinement by adding biphasic nanoparticles to spheroidal graphite cast iron.

The changes in eutectic morphology are attributed to the “nucleation promotion-growth suppression” synergistic mechanism of EASiCp:

(1) Nucleation promotion: The small-sized SiCp in EASiCp directly act as nucleation cores; the larger-sized SiCp react to form carbon-rich regions, providing more nucleation sites; the increase in undercooling also raises the nucleation rate.
(2) Growth suppression: EASiCp suppresses grain growth through the Zener pinning effect. The pinning force is given by the formula [31]:

(6) $$F_{\mathrm{res}}=\frac{3f\cdot \gamma }{2r}$$

In the equation: *F*_res_ represents the constraint force; *f* denotes the volume fraction of second-phase particles; *r* stands for the radius of second-phase particles; *γ* refers to the specific surface energy of the interface.

The limiting grain growth size $D_{L}=\frac{4r}{3f}$ indicates that the greater the addition amount of EASiCp, the stronger the constraint force on grain growth, and the smaller the grain size.

### 3.5. Effect on Mechanical Properties at Room Temperature

**Figure 14:**
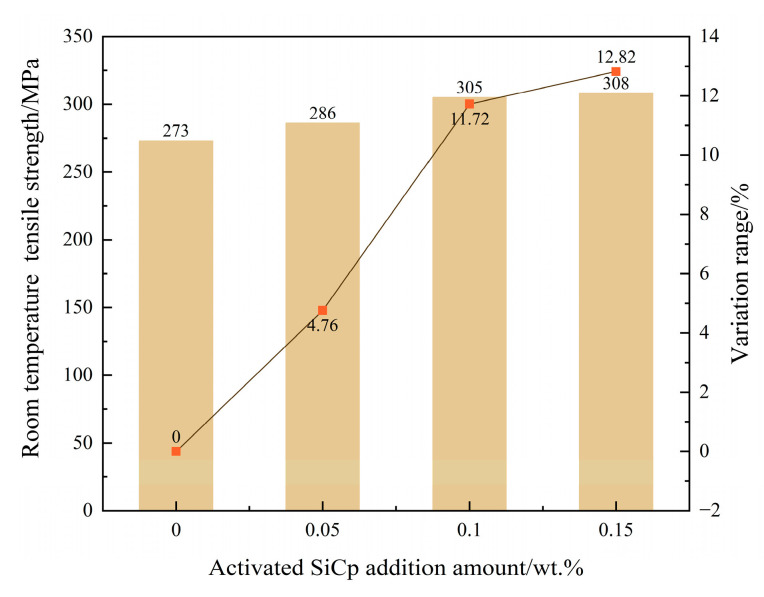
Effect of EASiCp Addition on Room-Temperature Tensile Strength of Hypoeutectic Gray Cast Iron (Error Bars Represent Standard Deviation of Three Measurements).

**Figure 15:**
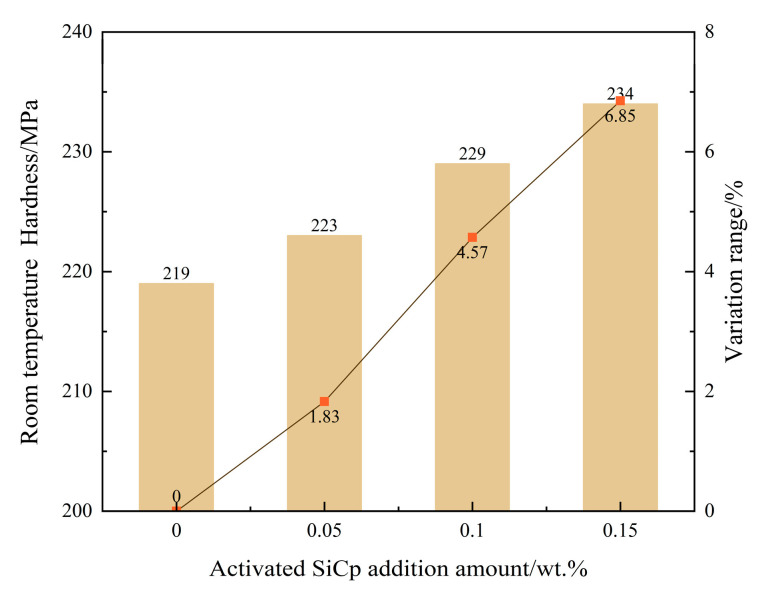
Effect of EASiCp Addition on Room-Temperature Hardness of Hypoeutectic Gray Cast Iron (Error Bars Represent Standard Deviation of Three Measurements).

To systematically evaluate the regulatory effect of EASiCp on the mechanical properties of hypoeutectic gray cast iron, this study tested the room-temperature tensile strength and Brinell hardness of the material at various addition levels. The results are shown in Figure 14 and Figure 15. The mechanical property test data indicates that with the increase in the EA-SiC particle addition, both the tensile strength and hardness of the material exhibit a consistent monotonic increase.

Specifically, compared to the unmodified baseline sample (273 ± 4 MPa tensile strength, 219 ± 3 HB hardness), when 0.15 wt.% EASiCp were added, the tensile strength increased to 308 ± 5 MPa, showing a 12.82% increase, and the hardness rose to 234 ± 3 HB, a 6.85% increase. Notably, the increase in hardness is significantly lower than the increase in tensile strength. This phenomenon is closely related to the distinct physical natures of these two mechanical properties: tensile strength mainly characterizes the material’s resistance to macroscopic plastic deformation until fracture and is highly sensitive to factors that hinder dislocation motion over long distances, such as grain boundaries and second-phase particles. In contrast, hardness is more focused on the material’s resistance to local plastic indentation, which is related to short-range dislocation movement and the material’s yield behavior under complex stress states. In this study system, EASiCp, through grain refinement and second-phase particle strengthening, effectively hinder dislocation slip over long distances during the tensile process, thereby contributing significantly to the enhancement of tensile strength. However, in the local indentation deformation involved in the hardness testing, the deformed region is subjected to a complex three-dimensional compressive stress state. Additionally, the presence of the graphite phase in the matrix, which acts as a weak point in strength, partly accommodates local deformation, thus partially diminishing the effect of microstructural refinement on the hardness improvement.

The reinforcement trend observed in this study is consistent with the findings of Poluboyarov et al. [16] regarding the effects of SiC reinforcement in gray cast iron, and also aligns with the strengthening effect of SiC in steel [32]. The synergistic enhancement of mechanical properties is essentially a macro manifestation of the multiscale synergistic optimization of the previously discussed microstructure:

(1) Grain refinement as the dominant mechanism: The addition of EASiCp significantly refined the secondary dendrite arm spacing of the primary austenite dendrites and the eutectic structure size. According to the Hall-Petch relationship, grain refinement greatly increases the grain boundary density, which effectively hinders dislocation movement, thus synchronously enhancing both strength and hardness of the material.
(2) Graphite morphology optimization played a critical role: The introduction of EASiCp caused the graphite flakes to become smaller, more curved, and more uniformly distributed. This optimized morphology effectively reduced the stress concentration at the graphite tips, delayed crack initiation and propagation, and enhanced the load-bearing efficiency of the material.
(3) Second-phase particle strengthening had a significant contribution: The residual SiCp, dispersed within the grains and at grain boundaries, form high-density dislocation fields around them due to the differences in thermal expansion coefficients between the ferrite/pearlite matrix and the particles. These hard particles also serve as effective dislocation pinning points, collectively reinforcing the matrix of the material.

## 4. Conclusions

This study systematically investigates the regulatory role and mechanisms of high-energy mechanically activated silicon carbide particles (EASiCp) on the solidification process, microstructure, and mechanical properties of hypoeutectic gray cast iron. The main conclusions are as follows:

(1) Particle characteristics successfully optimized: The high-energy activation treatment significantly refined the SiCp, reducing the average particle size from 26.53 μm to 9.51 μm, and increasing the specific surface area from 0.35 m^2^/g to 1.78 m^2^/g. XRD analysis confirmed that after activation, the particle grain size was refined to about 17.4 nm, with significant lattice distortion, placing them in a high-activity state.
(2) Stable and controllable behavior in the melt: The EASiCp particles exhibited a stable absorption rate (68–72%) in the iron melt, with a particle-size-dependent dissolution behavior. Ultimately, the submicron particles (0.3–2 μm) were dispersed in the matrix, mainly located within the grains (78.12%) and at the grain boundaries (21.88%).
(3) Significant changes in solidification kinetics: The addition of EASiCp effectively regulated the solidification kinetic parameters, increasing the undercooling from 5.3 °C to 8.4 °C and significantly lowering the latent heat of crystallization from 162.6 J/g to 99.96 J/g. This was primarily due to its ongoing endothermic reaction (SiC + Fe → FeSi + C) and strong heterogeneous nucleation effects.
(4) Synergistic mechanism dominates the microstructural refinement: EASiCp optimizes the microstructure through a synergistic mechanism of “promoting nucleation and inhibiting growth.” At the optimal addition amount of 0.15 wt.%, the primary austenite dendrite content increased by 35.29%, and the secondary dendrite arm spacing decreased by 57.98%. The A-type graphite flake length reduced from 0.46 mm to 0.20 mm, and the eutectic structure size was refined from over 500 μm to approximately 180 μm. This mechanism is attributed to the particles’ direct nucleation effect, the additional nucleation sites provided by the “micro-region carbon enrichment” from the reaction products, and the pinning effect on grain boundary migration.
(5) Simultaneous enhancement of mechanical properties: The synergistic optimization of the microstructure directly translated into an improvement in mechanical properties, with the material’s room-temperature tensile strength reaching 308 MPa, a 12.82% increase compared to the unmodified sample. This performance enhancement results from the combined effects of grain refinement, dislocation strengthening, and graphite morphology optimization.

In conclusion, the introduction of EASiCp provides an efficient and controllable technical pathway for regulating the solidification process of hypoeutectic gray cast iron. The “nucleation-growth” synergistic regulation strategy not only significantly refines the microstructure and enhances mechanical properties, but also lays a solid theoretical and experimental foundation for the development of high-performance cast iron components. This study identified an optimal effect within a SiC addition concentration of 0.15 wt.%. However, there may be an upper limit to the SiC addition concentration, beyond which particle agglomeration and performance degradation could occur. Determining the best processing window will be key for future industrial applications.

## Figures and Tables

**Table 1:** Main Parameters of SiC Raw Material (wt.%).

Code	SiC Content	Free Carbon Content	Free Silicon Content	Fe_2_O_3_ Content
GC	≥98.5	≤0.25	≤0.1	≤0.3

**Table 2:** Basic Chemical Composition of Hypoeutectic Gray Cast Iron (wt.%).

Component	C	Si	Mn	S	Ni	Fe	CE
Content	3.2	1.8–2.0	0.75–0.85	0.06–0.10	<0.02	balance	3.8–4.0

**Table 3:** Measured Chemical Composition of Hypoeutectic Gray Cast Iron under Different EASiCp Addition Amounts (wt.%).

EASiCp Addition Amount	C	Si	Mn	P	S	Ni	Fe	CE
0	3.213	1.824	0.786	0.026	0.084	0.016	Balance	3.876
0.05	3.221	1.839	0.791	0.026	0.085	0.014	Balance	3.890
0.1	3.235	1.871	0.788	0.027	0.084	0.014	Balance	3.914
0.15	3.242	1.886	0.782	0.026	0.084	0.014	Balance	3.926

**Table 4:** Comparison of particle size distribution of SiC powder before and after high energy activation modification.

Sample	D (4, 3)	D3	D50	D25	D (3, 2)	D90	D97	S.S.A.
Original	26.53 μm	5.04 μm	24.54 μm	14.92 μm	17.07 μm	46.61 μm	60.79 μm	0.35 sq.m/c.
EASiCp	9.51 μm	0.76 μm	7.07 μm	3.15 μm	3.36 μm	21.78 μm	29.33 μm	1.78 sq.m/c.

**Table 5:** The Specific Data on the Particle Size Distribution of EASiCp After Treatment.

Particle Size/μm	Differential Distribution/wt.%	Cumulative Distribution/wt.%	Particle Size/μm	Differential Distribution/wt.%	Cumulative Distribution/wt.%	Particle Size/μm	Differential Distribution/wt.%	Cumulative Distribution/wt.%
0.20			2.89	3.06	23.01	38.00	0.87	99.66
0.24	0.00	0.00	3.50	4.41	27.42	41.84	0.14	99.80
0.29	0.07	0.07	4.24	5.82	33.24	50.00	0.20	100.00
0.35	0.20	0.27	5.00	5.32	38.56	50.64	0.00	100.00
0.43	0.44	0.71	6.21	7.18	45.74	54.00	0.00	100.00
0.52	0.60	1.31	7.51	6.41	52.15	61.28	0.00	100.00
0.63	0.77	2.08	9.09	6.80	58.95	74.17	0.00	100.00
0.76	0.93	3.01	10.00	3.73	62.68	89.76	0.00	100.00
0.92	1.67	4.68	13.53	11.78	74.46	108.63	0.00	100.00
1.11	2.77	7.45	16.11	6.25	80.71	131.47	0.00	100.00
1.35	4.24	11.69	19.50	6.13	86.84	159.11	0.00	100.00
1.63	3.29	14.98	23.60	5.46	92.30	192.57	0.00	100.00
1.97	2.50	17.48	28.56	4.13	96.43	233.06	0.00	100.00
2.39	2.47	19.95	34.57	2.36	98.79	282.06	0.00	100.00

**Table 6:** Retention Rate of EASiCp in Hypoeutectic Gray Iron Melt.

Content of EASiCp/wt.%	Measured Ni Content/wt.%	Actual Ni Increment/wt.%	Theoretical Ni Increment/wt.%	Absorption Rate/%
0	0.014	0	0	-
0.2	0.028	0.014	0.02	70
1	0.086	0.072	0.1	72
3	0.219	0.205	0.3	68.3
5	0.358	0.344	0.5	68.8

**Table 7:** EDS test results (Spectrum 44).

Element	Weight Percent/wt.%	Atomic Percent/%
C K	22.59	51.40
Si K	22.15	21.55
Mn K	0.65	0.32
Fe K	54.62	26.73
Total	100.00	

**Table 8:** Dissolution Rates of EASiCp with Different Initial Particle Sizes.

*d*_0_/μm	*d_t_*/μm	*t*/min	*v*/(μm·min^−1^)
28–50	2–5	8	3.3–5.6
5–28	1–2	8	0.5–3.3
1.5–5	<1	8	<0.5

## Data Availability

The original contributions presented in this study are included in the article. Further inquiries can be directed to the corresponding authors.

## Nomenclature

*D*	The average grain size
Δ*T*	The degree of undercooling
*T_ic_*	The extrapolated crystallization onset temperature
*T_p_*	The peak temperature
*v*	The dissolution rate of EASiCp
*d_0_*	The initial particle diameter before reaction
*dₜ*	The particle diameter after reaction for t minutes
*Sc*	The Schmidt number
$\bar{S}$	The austenite dendrite morphology
*A_t_*	The total area of austenite dendrite cross-sections
*n_t_*	The number of intercepted austenite dendrite cross-sections
*F_res_*	The constraint force
*f*	The volume fraction of second-phase particles
*r*	The radius of second-phase particles
*γ*	The specific surface energy of the interface.
*K*	The Scherrer constant
*λ*	The X-ray wavelength
*β*	The full width at half maximum
*θ*	The diffraction angle
*μ*	The viscosity of the molten iron
*ρ*	The density of the molten iron
*Dc*	The diffusion coefficient of carbon
